# Supplementary material for: Monosex Populations of the Giant Freshwater Prawn Macrobrachium rosenbergii—From a Pre-Molecular Start to the Next Generation Era
Source: Int J Mol Sci. 2023 Dec 13;24(24):17433. doi: 10.3390/ijms242417433 (PMC10743721; doi:10.3390/ijms242417433)

Supplementary data

S1. Protein sequences of IAG, insulin-like peptides and representative members of the insulin superfamily. The structural similarity within the insulin superfamily is demonstrated as follows: the signal peptide is highlighted in light blue. The B (first) and A (second) chains are accentuated with a green background, and predicted cleavage sites are marked in red. Conserved Cys residues are shown with a yellow, pink, and blue background, in which each color pair of two Cys residues represents a disulfide bond.

>ACJ38227.1 insulin-like androgenic gland specific factor (IAG) [Macrobrachium rosenbergii]

MGYWNAEIKCVLFCSLVASLLPQPSSSYEIECLSVDFDCGDITNTLASVCLRHNNYINPGPTYVSKERRSADI
YTVPSTKSPSLAHPRATHLTMADEETQKVSKVEEEIQHMTLSREEANNMLHSKRRFRRDSVRRSPREECC
NNASFRRCNFEEVAEYCIELRPGVNTCSSR

>ABH07705.1 insulin-like androgenic gland factor (IAG) [Cherax quadricarinatus]

MLFQTLLNLILVVVVKLPPPSASYRVENLLIDFDCGHLADTMDSICRTYQEFNDTRAVRSARDASFSASVSM
YDPGSKIAVRQVYHPRGRKLGVKFTVPDARLGKQEAMTVSREAAHTFIKTQNYNRRRRNSDTTDNTSSTN
VYDECCSEKTLKTCVFDEIAQYCEQLEDGIYVSS

>AHY99679.1 insulin-like androgenic gland specific factor (IAG) [Sagmariasus verreauxi]

MLAPILLKLVVLAGMRQLPAASYNVSGLSEDFECGDFENVLGRICAETQSNIVRDTRSVSTVAVADSTHGG
TDPSRRPYHHPRAIQVVLRHAANPPATQGAGAEEGVRVTSEAAFSLVKSRSIRDTRETNLQDECCPFPLVR
HCDKEEILHYCFLTEG

>Q9U8R2.1 Androgenic gland hormone (AGH) [Armadillidium vulgare]

MKGLVILVSLMCLALYNRICAYQVRGMRSDVLCGDIRFTVQCICNELGYFPTERLDKPCPWPNREKR*SAPE*
*DELAFEDYEDQDYFHPRALSIPSEIEHDNEKESDAFSILSRG*KREIAFYQECCNIRTEHKCNRTTVSLYCRTY

>P26729.1 Bombyxin-II (BXA6) [Bombyx mori]

MKILLAIALMLSTVMWVSTQQPQAVHTYCGRHLARTLADLCWEAGVDKR*SGAQFASYGSAWLMPYSEG*
*RG*KRGIVDECCLRPCSVDVLLSYC

>P01325.1 Insulin-1 [Mus musculus]

MALLVHFLPLLALLALWEPKPTQAFVKQHLCGPHLVEALYLVCGERGFFYTPKSRR*EVEDPQVEQLELGGSP*
*GDLQTLALEVARQ*KRGIVDQCCTSICSLYQLENYCN

>P07455.2 Insulin-like growth factor I (IGF1) [Bos taurus]

MGKISSLPTQLFKCCFCDFLKQVKMPITSSSHLFYLALCLLAFTSSATAGPETLCGAELVDALQFVCGDRGFY
FNKPTGYGSSSRRAPQTGIVDECCFRSCDLRRLEMYCAPLKPAKSARSVRAQRHTDMPKAQKEVHLKNTS
RGSAGNKNYRM

>P04808.1 Prorelaxin H1 (REL1) [Homo sapiens]

MPRLFLFHLLEFCLLLNQFSRAVAAKWKDDVIKLCGRELVRAQIAICGMSTWSKR*SLSQEDAPQTPRPVAEI*
VPSFINKDTETIIIMLEFIANLPPELKAALSERQPSLPELQQYVPALKDSNLSFEEFKKLIRNRQSEAADSNPSE
*LKYLGLDTHSQ*KKRRPYVALFEKCCLIGCTKRSLAKYC

>AIU40994.1 insulin-like peptide 2 [Sagmariasus verreauxi]

MRTVGALVLVVVLAAAMVETRPYEETRSYKICTSRDVKVMANYVCNLHRRRRSVLSLDDARDNYGVPGLL
LENRSRRLALPQHWRPEDDTDTGNVSRRDPSFLQFTRIRRQVLLGEIRKQCCVHGCTPRDFYGACQ

>AIU40993.1 insulin-like peptide 2 [Cherax quadricarinatus]

MGRCSLRGIGALVFVLATAALLVETRPPYRSRRGMKVCSPRDVKFMATYICNLHRRSVRSVDDFEDDFESP
GVSRLSGVNIPPWRPSRCGNTGRDCGPGLDSSSSLPPLARLHHHSPPAAATTITAADFNRWLSLNGYHDL
SLQEDSNGLGNDLVNDPWQAIRENGETNQERENGLVRVNSATHFLGDISLAKRDREVDWPVLVPRSLSDI
RRNCCLRECTAEDFYGACS

S2. Multiple sequence alignment of the IAG and representative members of the insulin superfamily (sequences in S1) shows a typical proteomic structure of B and A chains with conserved Cys residues (brown background) linked by disulfide bonds (grey lines).

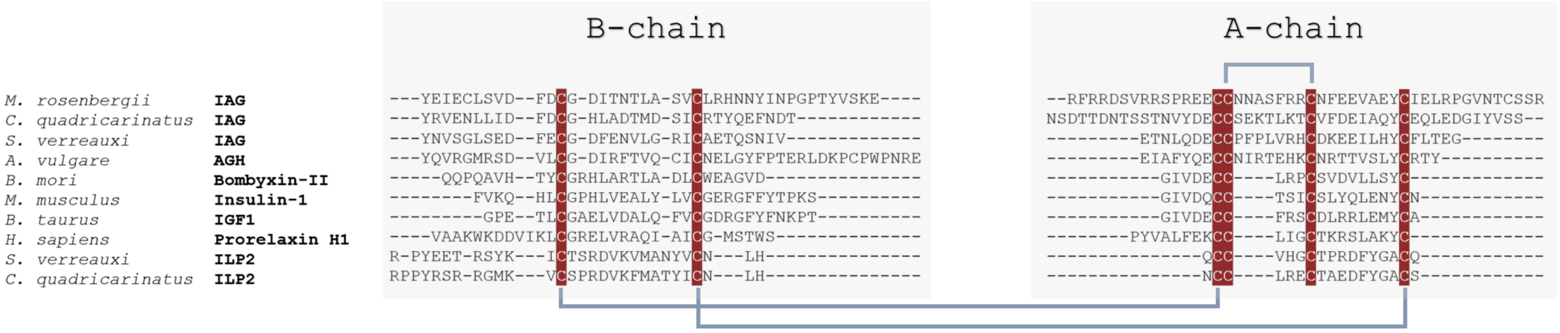

Supplement: Supplementary file 1 [file ijms-24-17433-s001.zip › ijms-2386877-supplementary.pdf]
